# Being First Matters: Topographical Representational Similarity Analysis of ERP Signals Reveals Separate Networks for Audiovisual Temporal Binding Depending on the Leading Sense

**DOI:** 10.1523/JNEUROSCI.2926-16.2017

**Published:** 2017-05-24

**Authors:** Roberto Cecere, Joachim Gross, Ashleigh Willis, Gregor Thut

**Affiliations:** ^1^Centre for Cognitive Neuroimaging, Institute of Neuroscience and Psychology, University of Glasgow, Glasgow G12 8QB, United Kingdom, and; ^2^Centre for Neuroscience, Strathclyde Institute of Pharmacy and Biomedical Sciences, University of Strathclyde, Glasgow G4 0RE, United Kingdom

**Keywords:** audiovisual integration, ERPs, representational similarity analysis, simultaneity judgments, temporal binding, topographical analysis

## Abstract

In multisensory integration, processing in one sensory modality is enhanced by complementary information from other modalities. Intersensory timing is crucial in this process because only inputs reaching the brain within a restricted temporal window are perceptually bound. Previous research in the audiovisual field has investigated various features of the temporal binding window, revealing asymmetries in its size and plasticity depending on the leading input: auditory–visual (AV) or visual–auditory (VA). Here, we tested whether separate neuronal mechanisms underlie this AV–VA dichotomy in humans. We recorded high-density EEG while participants performed an audiovisual simultaneity judgment task including various AV–VA asynchronies and unisensory control conditions (visual-only, auditory-only) and tested whether AV and VA processing generate different patterns of brain activity. After isolating the multisensory components of AV–VA event-related potentials (ERPs) from the sum of their unisensory constituents, we ran a time-resolved topographical representational similarity analysis (tRSA) comparing the AV and VA ERP maps. Spatial cross-correlation matrices were built from real data to index the similarity between the AV and VA maps at each time point (500 ms window after stimulus) and then correlated with two alternative similarity model matrices: AV_maps_ = VA_maps_ versus AV_maps_ ≠ VA_maps_. The tRSA results favored the AV_maps_ ≠ VA_maps_ model across all time points, suggesting that audiovisual temporal binding (indexed by synchrony perception) engages different neural pathways depending on the leading sense. The existence of such dual route supports recent theoretical accounts proposing that multiple binding mechanisms are implemented in the brain to accommodate different information parsing strategies in auditory and visual sensory systems.

**SIGNIFICANCE STATEMENT** Intersensory timing is a crucial aspect of multisensory integration, determining whether and how inputs in one modality enhance stimulus processing in another modality. Our research demonstrates that evaluating synchrony of auditory-leading (AV) versus visual-leading (VA) audiovisual stimulus pairs is characterized by two distinct patterns of brain activity. This suggests that audiovisual integration is not a unitary process and that different binding mechanisms are recruited in the brain based on the leading sense. These mechanisms may be relevant for supporting different classes of multisensory operations, for example, auditory enhancement of visual attention (AV) and visual enhancement of auditory speech (VA).

## Introduction

Imagine being at a busy intersection having a conversation with a friend while you both are about to cross the road. Because of the traffic noise, you look at your friend to better understand what s/he is saying without paying much attention to the cars that you see in the background. But when you suddenly hear that an engine noise is rapidly approaching, the situation changes and one of the cars that you ignored so far immediately catches all of your attention just in time to see its trajectory and avoid collision.

The situation described above exemplifies how both visual and auditory processing can benefit from complementary information coming from another sensory modality and illustrates two ways of combining audiovisual information to achieve the same goal: effectively interacting with the environment. However, although receiving two inputs from different sensory channels is necessary for such multisensory gain, it is not sufficient because appropriate timing is paramount to combine them in a unified percept. Absolute intersensory timing is important because perceptual binding only occurs within specific temporal binding windows (TBWs) (Meredith et al., 1987; Colonius and Diederich, 2004; van Wassenhove et al., 2007; Wallace and Stevenson, 2014), which can span up to hundreds of milliseconds depending on stimuli and tasks (van Eijk et al., 2008; Stevenson and Wallace, 2013). Moreover, the relative timing of multisensory inputs is also highly relevant, for example, whether a sound precedes a visual stimulus or vice versa. In visual-to-auditory (VA) interactions such as visual enhancement of auditory speech comprehension (Sumby and Pollack, 1954; Munhall et al., 2004; van Wassenhove et al., 2005), anticipatory visual information can facilitate the tracking of the dynamic auditory speech signal by providing useful predictive cues about the onset of syllables and words (Grant and Seitz, 2000; Chandrasekaran et al., 2009; Schwartz and Savariaux, 2014). Similarly, in auditory–visual (AV) interactions such as the enhancement of visual detection by sounds (Frassinetti et al., 2002; Bolognini et al., 2005; Van der Burg et al., 2008; Cecere et al., 2014), the alerting value of auditory cues would be lost if the correspondent visual event has already taken place. In this case, the much faster latencies of primary auditory than visual cortex activation (Murray and Spierer, 2009; Musacchia and Schroeder, 2009) may in principle enable auditory information to reach visual areas ahead of the incoming visual information and “alert“ the visual system to process it.

Conceptually, vision and audition seem therefore to benefit in different ways from crossmodal temporal cues in the multisensory context. Accordingly, some investigators have recently proposed that primary auditory and visual systems, which use different strategies to parse information (VanRullen et al., 2014), might also make different use of complementary information coming from other senses (Thorne and Debener, 2014). In other words, different mechanisms of crossmodal interaction might come into play depending on whether visual signals are cueing auditory processing (VA) or auditory signals are cueing visual processing (AV). Behavioral evidence indeed points in this direction. For instance, the time scale for evaluating audiovisual simultaneity depends on the temporal order of unisensory constituents, resulting in asymmetries in the TBW size (AV-TBW<VA-TBW) (Dixon and Spitz, 1980; Conrey and Pisoni, 2006; van Wassenhove et al., 2007) and plasticity (VA-TBW but not AV-TBW is trainable) (Powers et al., 2009; Cecere et al., 2016) depending on the leading sense.

Here, we recorded event-related potentials (ERPs) while participants performed an audiovisual simultaneity judgment task with different stimulus onset asynchronies (6 AV, 6 VA, 1 synchronous) to investigate whether the AV–VA dichotomy observed at the behavioral level reflects the activity of two separate brain networks. We used an additive model to remove unisensory components from AV and VA ERPs (AV = AV − (A + V); VA = VA − (V + A), taking into account differences in temporal alignment across conditions) and a time-resolved representational similarity analysis (RSA) (Kriegeskorte et al., 2008) to evaluate the dissimilarity between AV and VA topographies.

## Materials and Methods

### 

#### 

##### Participants.

Sixteen healthy volunteers gave written informed consent to participate in the study. All participants had normal hearing and normal or corrected vision by self-report and no history of neurological illness. Two participants were excluded from further testing due to inconsistent behavioral performance during the practice session and one participant failed to complete the EEG recording session. The remaining 13 participants (11 female; mean age: 21.9 years) were all right-handed.

All experimental procedures complied with the Declaration of Helsinki and were approved by the Ethics Committee of the College of Science and Engineering, University of Glasgow.

##### General procedure.

Each participant underwent two sessions over two consecutive days. On the first day of testing, participants practiced the behavioral task performing two blocks of 15 min each. During this pretest practice, participants were familiarized with the delayed response required by the task (see below) and encouraged to limit eye blinks to the manual response phase to reduce EEG artifact contamination in the following session. Data collected during the practice session were checked to ensure that participants produced a reliable behavioral performance before proceeding to the EEG session. On the second day of testing, participants performed a longer version of the behavioral task while EEG was recorded.

##### Behavioral apparatus and stimuli.

During both sessions, participants sat in a dimly lit, electromagnetically shielded room with their head stabilized by a chin rest. Visual and auditory stimuli were presented using E-Prime software (version 2.0). Manual responses were collected via a standard computer keyboard. Visual stimuli consisted of a 10 ms white annulus (external diameter: 9° of visual angle; inner diameter: 4.5°) surrounding a central fixation cross (1° × 1°) and were presented on a CRT monitor (100 Hz refresh rate) at 85 cm distance from the chin rest. Auditory stimuli were a 10 ms sinusoidal pure tone (frequency: 1800 Hz; sampling rate 44100 Hz) delivered at a sound pressure level of 75 dB via a speaker positioned at the bottom of the monitor.

##### Behavioral task.

**Figure 1.**
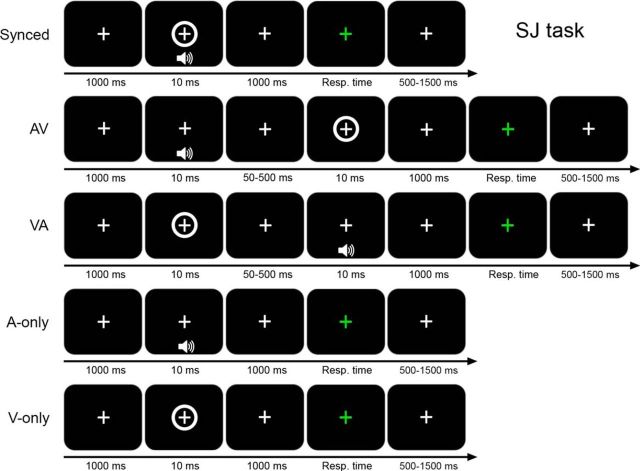
Schematic overview of the simultaneity judgment task. In multisensory trials (rows 1–3), auditory and visual stimuli were presented either in physical synchrony (Sync), or asynchronously, i.e., with the auditory stimulus leading the visual stimulus (AV) or with the visual stimulus leading the auditory stimulus (VA). In unisensory trials (rows 4–5), a single auditory (A-only) or visual (V-only) stimulus was presented.

The behavioral task used in both practice and EEG sessions of the experiment was an audiovisual simultaneity judgment (SJ) task with delayed response, requiring participants to evaluate the simultaneity of auditory and visual stimuli that could be presented either in perfect synchrony or with a variable stimulus onset asynchrony (SOA) (Fig. 1). The delayed response protocol was implemented to avoid contamination of the poststimulus EEG signal by the motor response.

Each trial began with a central fixation cross (1000 ms duration) on a gray background, followed by auditory and/or visual stimulus presentation, in one of 15 possible conditions: 1 audiovisual synchronous condition (Sync; 0 ms SOA), 12 audiovisual asynchronous conditions (±50, ±100, ±150, ±200, ±250, and ±500 ms SOA; minus sign: AV conditions; plus sign: VA conditions) and two unisensory conditions (auditory only or visual only). After stimulus presentation, participants had to refrain from giving their manual response for 1000 ms until they received a response cue consisting of the white fixation cross turning green. Participants then pressed “1” for “synchronous” or “2” for “asynchronous” on the keyboard using their right index and middle finger, respectively. If a unisensory trial (auditory or visual only) was presented, participants were instructed to press any button to move to the next trial. After the response, the green cross turned back to white and a new trial began after a variable intertrial interval (500–1500 ms).

A single block of the SJ task consisted of 15 repetitions of each of the 15 conditions (225 total trials), which were presented randomly. The practice session consisted of two such blocks (30 trials × 15 conditions = 450 total trials) with a break in between. The EEG session consisted of four blocks (60 trials × 15 conditions = 900 total trials) with a break after each block.

##### EEG acquisition and ERP preprocessing.

High-density EEG was recorded from 128 Ag/AgCl scalp electrodes mounted in an elastic head cap (BioSemi). The analog signal was digitized at 1024 Hz and amplified using an ActiveTwo system (BioSemi).

Raw EEG data were preprocessed using the EEGLAB toolbox (Delorme and Makeig, 2004) running under MATLAB (The MathWorks). For each participant, the continuous EEG signal was filtered offline (high-pass: 1 Hz, low-pass: 40 Hz) and segmented in 2000 ms epochs spanning between 1000 ms before and 1000 ms after the onset of the first stimulus in the audiovisual pair. After epoching, data were inspected visually to identify and remove contaminated EEG signals. Bad channels were removed (on average, 2.8 ± 1.9% of 128 channels), but not interpolated at this stage, and epochs contaminated by artifacts were rejected (4.6 ± 2.5% of trials, leaving at least 56 of 60 trials per condition) using an extreme value rejection criterion of ±50 μV. After rereferencing all data to the channel average, an independent component analysis (Delorme and Makeig, 2004; Delorme et al., 2007) was used to identify and remove artifacts related to blink activity from the EEG and previously removed channels were interpolated using a spherical spline interpolation. Subsequently, artifact-free trials were extracted for each of the 15 conditions (Sync, AV50, AV100, AV150, AV200, AV250, AV500, VA50, VA100, VA150, VA200, VA250, VA500, A-only, and V-only) and multisensory trials only (Sync, AV, and VA conditions) were reepoched from −500 ms to 1000 ms prestimulus to poststimulus-1 onset and baseline corrected (−500 ms to 0 ms). A-only and V-only trials were not reepoched yet at this stage, but were used to create synthetic (i.e., summed) multisensory ERPs later in the analysis pipeline, as detailed below.

##### Data analysis pipeline.

**Figure 2.**
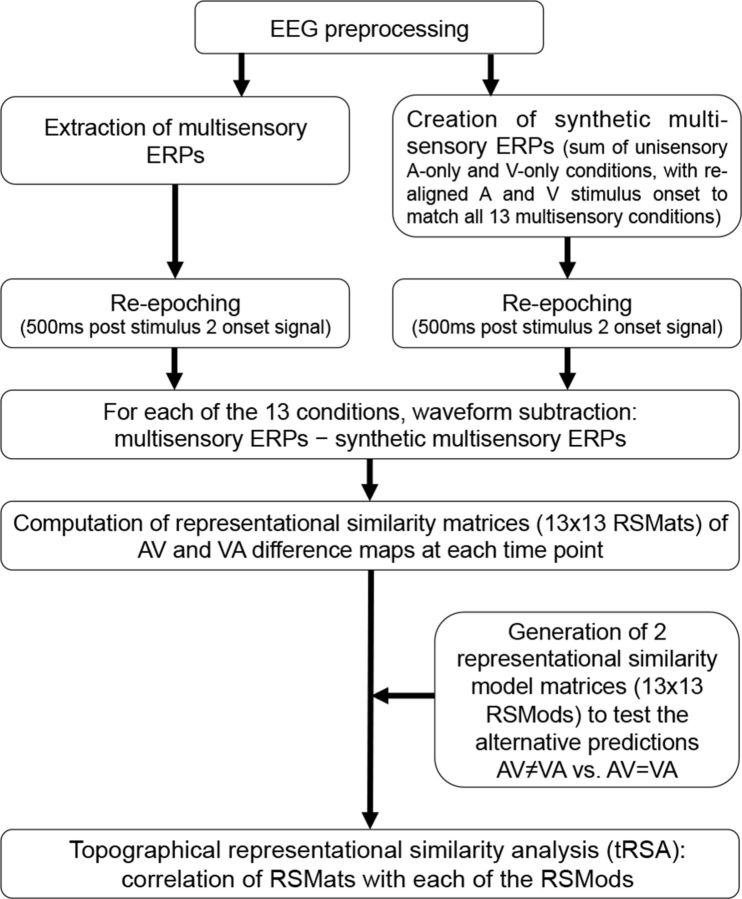
EEG data analysis pipeline.

The ERP analyses sought to establish whether perception of synchrony (i.e., temporal binding) of AV versus VA stimulus pairs is mediated by common or separate networks and, by extension, one single or multiple mechanisms. To achieve this, we used a topographical ERP analysis approach using spatial correlation coefficients (Murray et al., 2008) in combination with RSA (Kriegeskorte et al., 2008). In this analysis, hereafter referred to as topographical RSA (tRSA), we first used spatial correlation coefficients to quantify the degree of overlap (similarity) between brain activity patterns (ERP scalp maps) across conditions. Subsequently, we computed representational similarity matrices (RSMats) obtained from these spatial cross-correlation coefficients and compared them with either of two alternative models (identical or different maps for AV vs VA pairs). This approach (pipeline summarized in Fig. 2) allowed us to assess whether AV and VA processing elicits distinct, noncorrelated activation patterns (model 1: dual-network hypothesis) or, alternatively, that they generate similar, highly correlated topographic maps (model 2: single network/common multisensory nexus hypothesis). Custom MATLAB scripts were used to perform all analyses from this stage onward.

The analysis pipeline involved four main steps applied to the preprocessed EEG data (see above). In step 1, a subtractive method was used to remove unisensory components from the ERPs and isolate the nonlinear multisensory effects of interest (for examples of studies using this additive model, see Giard and Peronnet, 1999; Foxe et al., 2000; Raij et al., 2000; Molholm et al., 2002; Barth and Brett-Green, 2004; Besle et al., 2004; Calvert and Thesen, 2004; Murray et al., 2005; Senkowski et al., 2007; Talsma et al., 2007; Senkowski et al., 2011). This step was fundamental to make AV and VA conditions directly comparable, accounting for differences in the temporal order of the unisensory constituents of AV and VA pairs. For each participant, 13 ERPs were extracted from multisensory trials (6 AV, 6 VA, and 1 Sync) and as many synthetic “multisensory” ERPs were created by computing the algebraic sum of unisensory visual and auditory ERPs after realigning A and V stimulus onsets to match real multisensory SOAs. Subsequently, difference waveforms were computed for each condition by subtracting the synthetic ERPs from the real multisensory ERPs (for details, see “Step 1: Multisensory, unisensory sum and difference ERPs”). In step 2, the similarities of topographies across the 13 conditions were quantified at each time point via spatial correlation coefficients and RSMats. Specifically, at each time point, an RSMat was obtained by cross-correlating the map topographies of all 13 conditions with each other, resulting in a time series of RSMs each containing 13*13 spatial correlation coefficients (for details, see below: “Step 2: Spatial correlations and computation of representational similarity matrices”). In step 3, two representational similarity model matrices (RSMods) were generated, each corresponding to one of the two alternative predictions we sought to test (i.e., AV_maps_ ≠ VA_maps_ vs AV_maps_ = VA_maps_; see “Step 3: Generation of RSMods”). Finally, in step 4, the RSMats of actual ERP topographies were compared directly with the RSMods to test which model of spatial correlations best fitted real data (for a similar approach, see Kriegeskorte et al., 2008). This was done by correlating the RSMat at each time point with each of the two RSMods (see “Step 4: tRSA”).

##### Step 1: Multisensory, unisensory sum, and difference ERPs.

**Figure 3.**
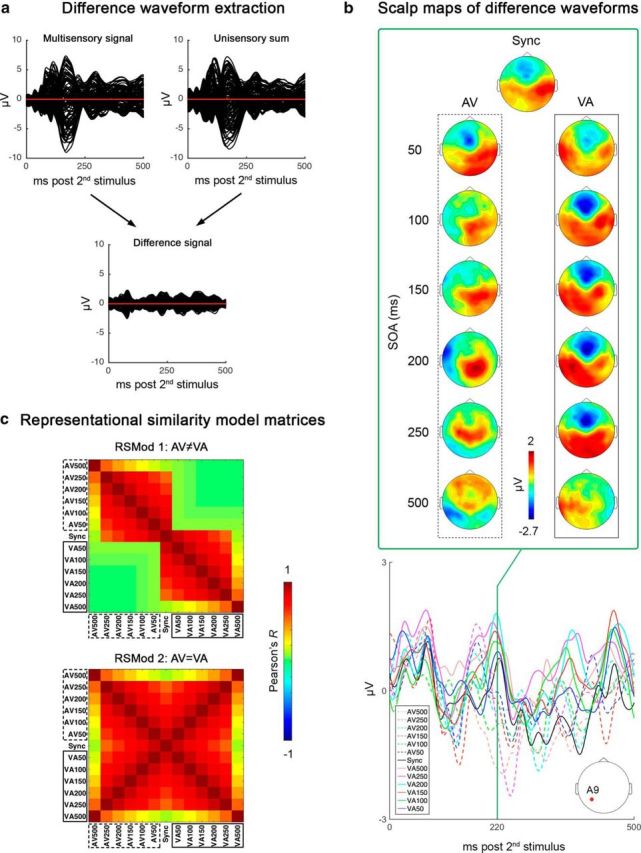
***a***, Examples of waveforms (all electrodes superimposed) extracted from multisensory (top left) and summed unisensory (top right) trials (here illustrated for the Sync condition). The difference waveform (bottom) obtained by subtracting unisensory components from multisensory signals represents the primary signal of interest (multisensory processing). ***b***, Examples of difference scalp topographies (at 220 ms) and waveforms (A9 electrode, 0–500 ms) for all 13 multisensory conditions. Time-point zero corresponds to the onset of stimulus-2 of the audiovisual pair. The waveform plot indicates that, around 220 ms, the ERP amplitudes evoked by AV stimulus pairs (dotted lines) maximally diverge from VA pairs (solid lines). The corresponding maps show similar patterns of activation within but not between AV and VA conditions. ***c***, Representational similarity model matrices of the dual network hypothesis (RSMod 1: AV ≠ VA) and of the single network hypothesis (RSMod 2: AV = VA) for audiovisual temporal integration. RSMod 1 predicts different topographic maps between AV and VA conditions (i.e., high correlation within but not between AV and VA). RSMod 2 predicts no difference between maps generated by AV and VA conditions (i.e., high correlation within and between AV and VA conditions).

For each participant, ERPs were extracted for the 13 multisensory conditions (i.e., per SOA) by averaging multisensory trials (−500 ms to +1000 ms prestimulus to poststimulus-1 onset). To create matching synthetic multisensory ERPs, unisensory A-only and V-only ERPs (initially −1000 ms to +1000 ms prestimulus to poststimulus) were first reepoched with different prestimulus and poststimulus intervals to align stimulus onsets (visual or auditory) to each of the 13 real multisensory conditions/SOAs and then summed. For example, to obtain a synthetic ERP corresponding to the AV100 condition, A-only ERPs were reepoched from −500 to +1000 ms, whereas V-only ERPs were reepoched from −600 to +900 ms such that, when summed, the auditory and visual stimulus onset would be separated by 100 ms. After summing the unisensory waveforms, the new synthetic ERPs (now −500 ms to +1000 ms prestimulus to poststimulus-1 onset) were baseline corrected using the same prestimulus interval as the real multisensory ERPs (−500 to 0 ms). Once both multisensory ERPs and their synthetic (unisensory sum) counterpart were obtained, they were reepoched to extract the 500 ms window poststimulus-2 onset and finally subtracted from each other (multisensory − unisensory sum) (for examples, see Fig. 3*a*) to obtain difference waveforms/topographies of the 13 audiovisual conditions (for examples, see Fig. 3*b*). We focused on the 500 ms poststimulus-2 window because our analysis aimed to compare topographic maps generated by AV versus VA multisensory processing, which can reasonably take place only after the presentation of the second stimulus of the audiovisual pair. Accordingly, all subsequent analyses were performed on the 500 ms residual signal resulting from the above procedure.

##### Step 2: Spatial correlations and computation of RSMats.

After extracting the difference signal of interest, Pearson's spatial cross-correlation coefficients (*R*) were used to index the degree of similarity among all AV, VA, and Sync scalp maps at each point of the time series (equivalent to spatial correlation coefficients used for ERP map analysis in Murray et al., 2008). For all participants and conditions, voltage maps at each time point were first redefined as 21-point average maps using a moving window (window size: 21 time points = ∼20 ms; window center moving in 1-time-point steps). Then, time-point-wise correlation indices were calculated across the maps of the 13 conditions, resulting in a 13*13 RSMat per time point and participant and each representing a “snapshot” of the cross-correlation pattern between AV and VA maps at each time point.

##### Step 3: Generation of RSMods.

**Figure 4.**
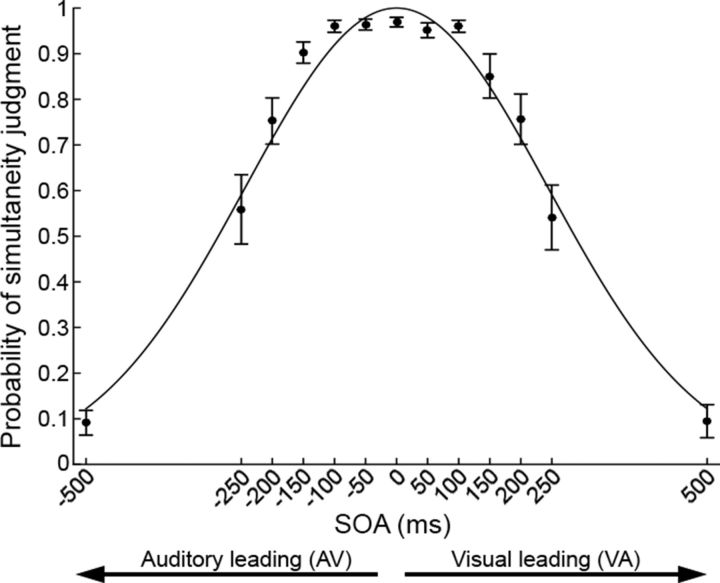
Behavioral results. Shown is a plot of the probability of perceiving audiovisual simultaneity as a function of SOA (black dots) and best-fitting Gaussian function (black line). Error bars indicate SEM.

After computing the RSMats from real data, two 13*13 RSMods were generated, reproducing the spatial cross-correlation patterns predicted by our two alternative hypotheses (AV ≠ VA vs AV = VA). The spatial correlation coefficients within the two RSMods were modeled on behavioral data (see “Behavioral data” in the Results section below and Fig. 4), expecting that the degree of correlation between topographies along SOAs would change in a similar fashion as the probability of perceived simultaneity in the SJ task. A Gaussian fit was performed on the behavioral psychometric curve (using the MATLAB curve fitting tool) and the best-fitting function (*R*^2^ = 0.98) was then used to model the *R* values of each RSMod.

Consistent with hypothesis 1, that AV and VA ERPs have different, uncorrelated scalp distributions (AV ≠ VA), RSMod 1 (Fig. 3*c*, top plot) consists of two clusters of high spatial correlation indices (*R*) within the AV and VA conditions (upper left and lower right quadrants, respectively), but low *R* values between conditions (upper right and lower left quadrants). For the two high-correlation clusters, the initially high *R* values around the main diagonal (set to *r* = 0.9) decay along rows and columns following the Gaussian function fitting behavioral data. For the remaining two noncorrelated clusters, correlation values are all equal to the lowest correlation value of each row/column.

Reflecting hypothesis 2, that AV and VA conditions share a common network (AV = VA) and elicit the same activity pattern independent of leading sense (common multisensory nexus), conditions in RSMod 2 (Fig. 3*c*, bottom plot) are highly correlated along both diagonals (correlating with themselves and the inverse stimulus pair of the same SOA) because the model predicts that the order of presentation of auditory and visual stimuli is irrelevant. Like RSMod 1, the spatial correlation indices of the RSMod 2 are characterized by a Gaussian-shaped decay from the diagonals along row and columns.

##### Step 4: tRSA.

To ascertain which model (RSMod 1: AV ≠ VA; RSMod 2: AV = VA) best fitted real data (RSMats), we performed tRSA (Kriegeskorte et al., 2008) consisting of the computation of second-order correlations (i.e., similarity of similarity matrices) to index the similarity between our similarity matrices and models (RSMats vs RSMods). To this end, after excluding the values of the main diagonals containing autocorrelations to avoid artificial inflation of values, time-point-wise Spearman's rank correlations (*r*_s_) were computed between each of the two RSMods and the time series of RSMats in our window of interest (500 ms poststimulus-2), thus obtaining two time series of *r*_s_ values indexing the goodness of fit of each model to the data over time.

##### Statistics.

The model-fitting procedure was performed at the single participant level to allow for statistical tests, which involved a series of paired *t* tests (i.e., at each time point) between the two goodness-of-fit curves resulting from the RSA to assess whether and when they significantly differed; that is, one model significantly better predicted real data than the other. To determine a significance threshold for our *t* values and to address the multiple-comparisons problem, we used the maximum statistic approach (Nichols and Holmes, 2002), a nonparametric test based on permutations. This consisted of performing multiple times (5000 iterations) a series of *t* tests comparing two randomly resampled goodness-of-fit curves over the time window of interest obtained by randomly permuting the labels of the two fitted models (RSMod 1 fit and RSMod 2 fit) for each participant and iteration. This resulted, at each new iteration, in a new series of *t* values determined by surrogate data. The maximum *t* value of each of these series was then extracted to create a null distribution of *t* maxima. The *t* value corresponding to the 97.5 percentile of this distribution was considered as the cutoff value above which the difference between AV ≠ VA and AV = VA model fits was significantly above chance (α < 0.05, two-tailed).

## Results

In the present study, we used high-density EEG to investigate whether perception of simultaneity of AV and VA events is achieved (at least partially) through separate neural networks. We recorded ERPs during a classic simultaneity judgment task in several conditions (AV and VA pairs presented with 13 SOAs) and used a topographic representational similarity analysis approach using spatial cross-correlations and model fitting to compare scalp distributions of AV and VA ERPs. Analyses were performed on the multisensory responses after removing the constituent unisensory signals. Under the hypothesis that different neural networks and mechanisms are involved in audiovisual temporal binding depending on the leading sense, quantitatively different topographic ERP patterns are expected to emerge when participants judge synchrony between AV and VA multisensory events.

### Behavioral data

The behavioral data of the EEG session (Fig. 4) were entered in a 2 × 6 repeated-measures ANOVA with leading sense (AV or VA) and SOA (50, 100, 150, 200, 250, or 500 ms) as within-subject factors. As expected, the mean probability of perceived simultaneity decreased as a function of SOA (main effect of SOA: *F*_(5,60)_ = 157.18, *p* < 0.001). This demonstrates that participants were paying attention to the task and correctly executing it during EEG recording. No main effect of leading sense (*F*_(1,12)_ = 0.34, *p* = 0.57) and no interaction between leading sense and SOA were observed (*F*_(5,60)_ = 0.5, *p* = 0.77).

### EEG data

For the topographical analysis, we computed 128-channel ERPs reflecting audiovisual processing in 13 conditions differing in terms of leading sense (AV or VA) and SOA (50, 100, 150, 200, 250, or 500 ms). We then extracted the cross-correlation patterns of the ERP maps of all conditions (creating 13*13 RSMats) and used tRSA to compare these patterns with those predicted by two alternative cross-correlation models (RSMod 1: AV ≠ VA; RSMod 2: AV = VA).

### AV and VA simultaneity judgments elicit two distinct patterns of activation

**Figure 5.**
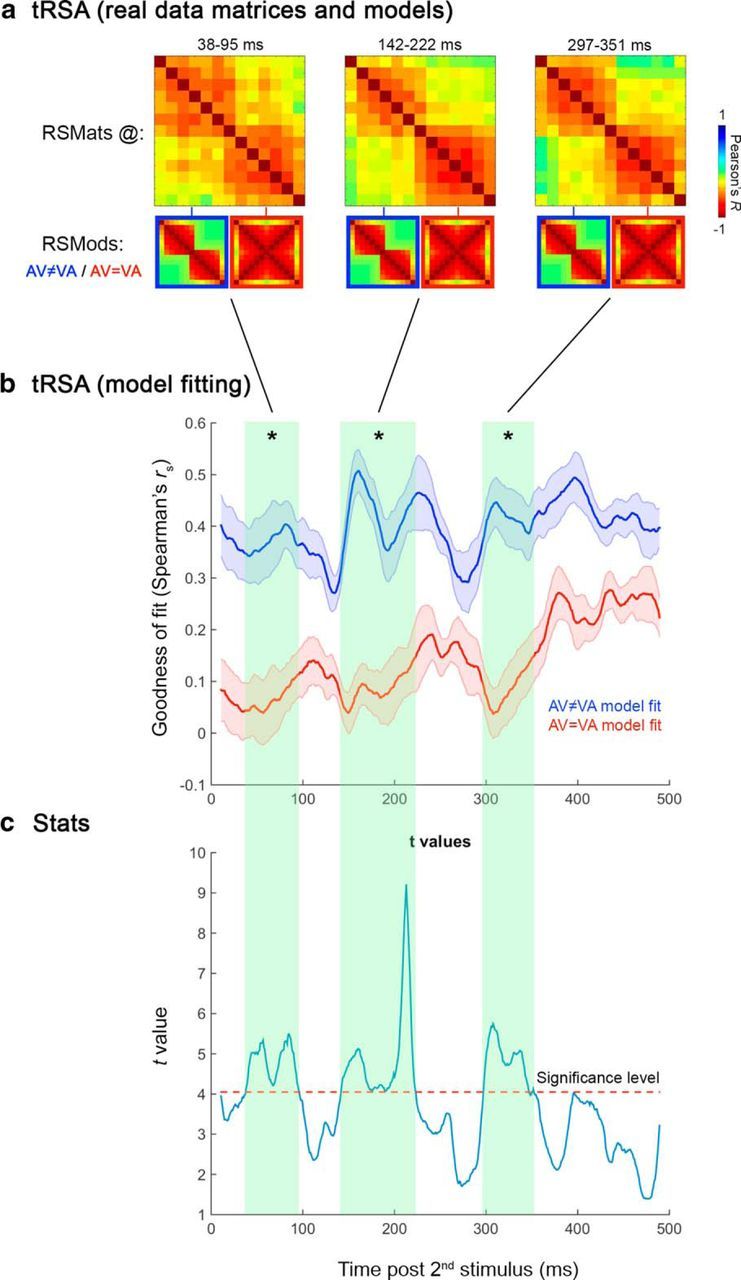
Results of the tRSA, in which two alternative RSMods of AV and VA topographies were fitted (i.e., correlated) to the RSMats obtained from real data at each time point of the 500 ms window poststimulus-2 onset. ***a***, Examples of RSMats obtained from real data (average over the 39–95 ms, 142–222 ms, and 297–351 ms time windows) and RSMods representing the AV ≠ VA (blue-framed) and AV = VA (red-framed) hypotheses. ***b***, Mean (*n* = 13 participants) goodness of fit (*r*_s_) of RSMod 1 (AV ≠ VA, blue line) and RSMod 2 (AV = VA, red line) to real data (RSMats) over time. Shaded error bars indicate SEM. ***c***, Results of the *t* test between the two model fits. The dashed red line marks the significance level (α = 0.05; two-tailed). Time intervals with significant differences between the two model fits are highlighted in aqua green and marked by asterisks. The AV ≠ VA RSMod was a significantly better predictor (higher goodness of fit to RSMats) than the AV = VA RSMod at 39–95 ms, 142–222 ms, and 297–351 ms poststimulus-2. The average RSMats over these three time windows are shown in ***a***. Note the striking similarity between RSMats and AV ≠ VA RSMod (blue-bordered).

The tRSA results are illustrated in Figure 5. The line plot (Fig. 5*b*) represents the fits of the AV ≠ VA RSMod (blue line) and the AV = VA RSMod (red line) to the data, expressed as correlation coefficient *r*_s_, over the whole 500 ms window poststimulus-2 onset. It is clear from the figure that the model predicting different maps elicited by AV and VA simultaneity judgments (AV ≠ VA RSMod) is by far better fitting the real data (mean *r*_s_ = 0.4) than the model of no difference between AV and VA maps (AV = VA RSMod; mean *r*_s_ = 0.14). The *r*_s_ values corresponding to the two RSMod fits (blue vs red line) were statistically compared time point by time point using dependent-sample *t* tests. Figure 5*c* (blue line) shows the plot of *t* values resulting from this analysis. The dashed red line represents the significance level (α = 0.05, two-tailed) as determined by permutation-based statistics accounting for multiple comparisons (maximum statistic; Nichols and Holmes, 2002). This analysis yielded three time intervals in which the AV ≠ VA model was a significantly better predictor than the alternative AV = VA model: 39–95 ms, 142–222 ms, and 297–351 ms (highlighted in aqua green in Fig. 5*b,c*). The three average RSMats (real data) corresponding to the significant time intervals are shown in Figure 5*a*. It is worth noting their striking similarity to the AV ≠ VA RSMod shown in both Figure 5*a* (blue-bordered matrices) and Figure 3*c* (top matrix). At no time points did the alternative RSMod (AV = VA) explain the real data better than the AV ≠ VA RSMod.

### Differences between AV and VA maps are not driven by differences in ERP latency

Further analyses were performed to ensure that AV and VA maps are genuinely different and that the above results were not biased by our approach consisting of time-point-wise correlations of AV and VA maps. Indeed, the fact that a topographic map of a given stimulus pair (e.g., AV) is different from the one elicited by the opposite pair (e.g., VA) at the same time point does not rule out that the two stimulus pairs may yet elicit the same topography but with different latencies. To rule out this confound, we ran a new spatial correlation analysis comparing the topographies that were specific to a stimulus pair with all other conditions not only at the same time point (i.e., as in the previous analysis), but also across the whole time series. Note that such a comparison did not entail building RSMats as in the main analysis, but rather involved the computation of spatial correlation coefficients between a given template map (one per window and condition) and the maps of all the other conditions over time (for a similar approach, see Murray et al., 2008). The Sync and AV–VA500 conditions were not included in this analysis, the former because it lacks the element of audiovisual delay, the latter because it yields extremely low behavioral perception of synchrony (Fig. 4) indicating low-to-no audiovisual temporal binding.

**Figure 6.**
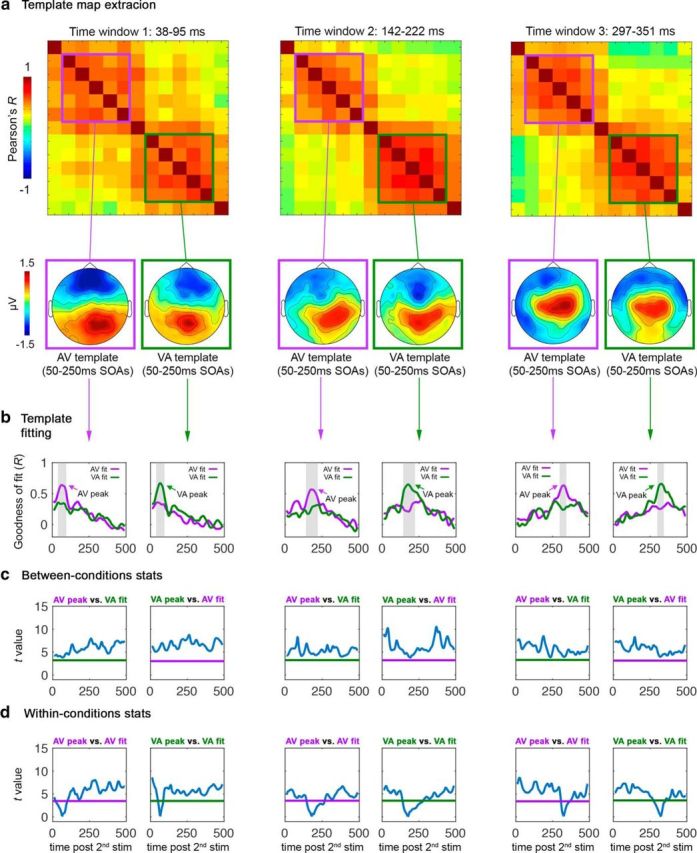
Results of the spatial correlation control analysis testing for reoccurrence of AV- and VA-specific maps at different latencies/conditions. ***a***, Template map extraction. The top shows the average RSMats over the three time windows in which AV and VA maps significantly differed in the main analysis (39–95 ms, 142–222 ms, and 297–351 ms). AV- and VA-specific maps of these three RSMats/time windows were extracted and used as templates in the spatial correlation analysis. In the bottom, below each RSMat, the corresponding clustered (average) AV and VA template maps are shown as examples, but the template map fitting was performed on single conditions using unclustered data (see Fig. 7 for separate templates per condition). ***b***, Template fitting results. For each time window, each AV and VA template map was fitted separately (correlated) to AV and VA maps/conditions over the 500 ms window poststimulus-2. Each plot displays the grand average goodness of fit of template maps to AV (purple lines) and VA (green lines) maps/conditions (SOAs collapsed) over time (see Fig. 8 for separate plots per condition). ***c***, Results of between-conditions *t* tests comparing each AV–VA peak *R* value in ***b*** with the *R* values of the opposite conditions fit (VA–AV). ***d***, Results of within-conditions *t* tests comparing each AV–VA peak *R* value in ***b*** with the other *R* values of the same fit. In both ***c*** and ***d***, the plots represent *t* values (blue lines) as a function of time. Purple and green horizontal lines represent the significance level (α = 0.05) for the AV and VA conditions, respectively. All *t* values above the significance level indicate that the peak *R* values marked by an arrow in ***b*** are significantly higher than the corresponding time points of the same fit curve; that is, the template map on top of that column is specific to the conditions/time point indicated by peaks in ***b*** and does not reoccur at any other time point condition.

**Figure 7.**
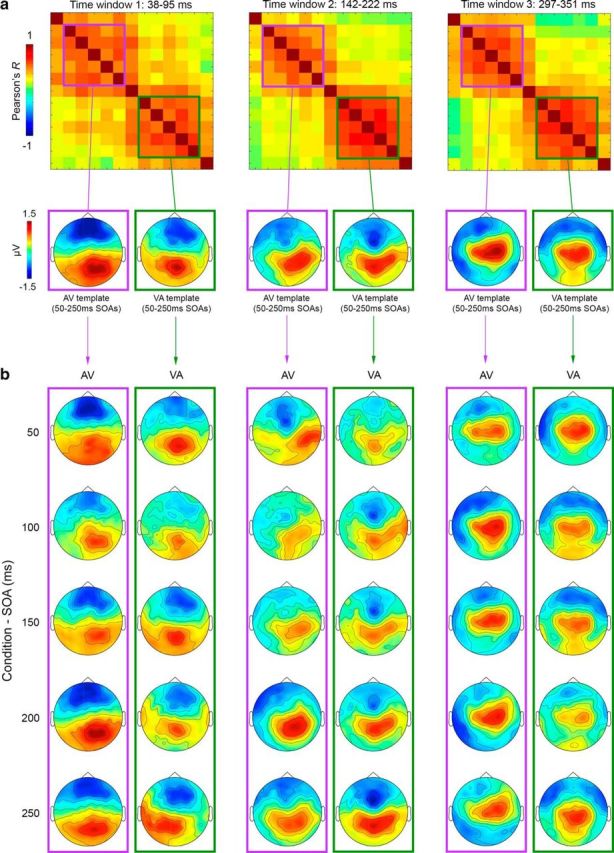
AV and VA maps used as templates in the map-fitting procedure (single conditions). ***a***, Topographical RSMats corresponding to the three time windows of interest (39–95 ms, 142–222 ms, and 297–351 ms; top) and average AV (purple-squared) and VA (green-squared) template maps corresponding to AV and VA clusters in the RSMats (bottom). ***b***, ERP maps of single AV (purple-squared) and VA (green-squared) conditions/SOAs, averaged over time window 1 (columns 1–2), time window 2 (columns 3–4), and time window 3 (columns 5–6).

**Figure 8.**
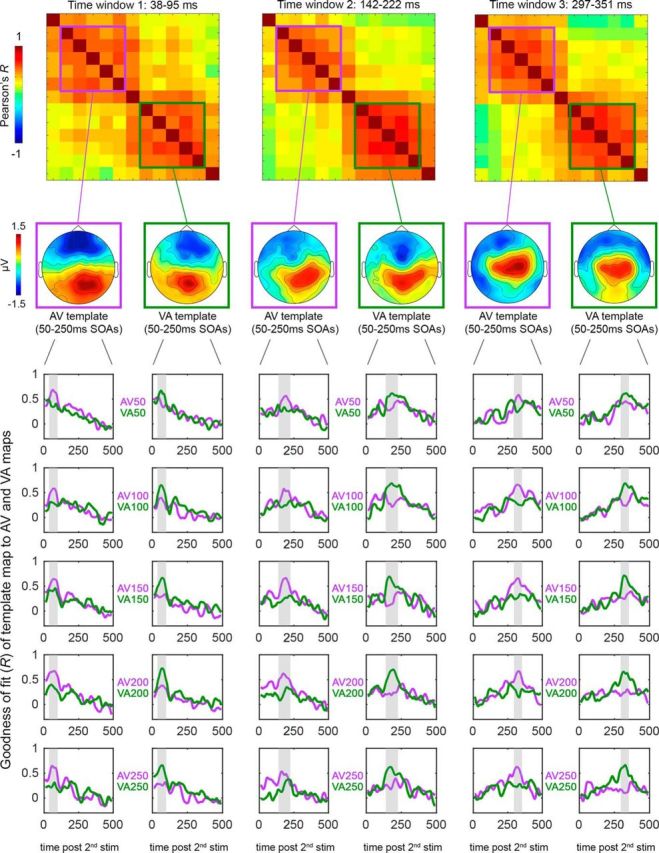
Template-fitting results (single conditions). Top, Topographical RSMats corresponding to the three time windows of interest (39–95 ms, 142–222 ms, and 297–351 ms). Below each RSMat are represented the average AV (purple-squared) and VA (green-squared) template maps corresponding to AV and VA clusters in the RSMats. The line plots show the fit of each template (i.e., the map at the top of each column) to the single AV (purple lines) and VA (green lines) conditions (50–250 ms SOAs). Note that the single-condition plots parallel the average results presented in Figure 6, showing that each AV (purple) and VA (green) template is highly correlated (see peak *R* values) only to same-group conditions (AV: purple lines; VA: green lines).

For each participant and time window in which AV and VA significantly differed in the main analysis (39–95 ms, 142–222 ms, and 297–351 ms), we first extracted the template maps for each of the five AV and VA conditions (AV50–250 and VA50–250, respectively) by averaging maps over each time window. By this means, we obtained five AV template maps (AV50-AV250) and five VA template maps (VA50-VA250) per time window of interest and participant. For ease of display, Figure 6*a* (bottom) shows grand-averaged template maps collapsed within AV (purple-squared) and VA (green-squared) clusters of high correlation coefficients (i.e., collapsed across SOAs) per time window of interest. Collapsing of AV and VA maps within clusters is motivated by their high cross-correlations and thus topographical similarity (for maps of each SOA before averaging, see Fig. 7). Subsequently, each of the extracted template maps was fitted (i.e., correlated) to all AV and VA maps/conditions over the whole time series (i.e., not only at the three significant time windows) to test whether any of the template maps (AV or VA) reoccur in a different condition/latency, as would be indicated by high topographical similarity (*R*) with maps of other condition/time point. The results of this template map fitting are summarized in Figure 6*b*, in which the plots show goodness of fit (*R*) of each AV and VA template map to AV (purple lines) and VA (green lines) conditions (averaged across all single AV and VA conditions for ease of display; see Fig. 8 for fits to single AV and VA conditions/SOAs). Figure 6*b* (see also Fig. 8) shows that AV and VA template maps best fit their respective condition group (i.e., AV templates maximally correlate with AV conditions and VA templates with VA conditions), but never reoccur (peak) in the opposite condition. For example, the 39–95 ms AV template map in Figure 6*a* (purple-squared) maximally correlates with AV conditions (see purple line in the corresponding plot of Fig. 6*b*) at 39–95 ms (see peak), but does not show any peak in the VA conditions (green line in the same plot). Conversely, the 39–95 ms VA template map in Figure 6*a* (green-squared) maximally correlates with VA conditions (see green line in the corresponding plot of Fig. 6*b*) at 39–95 ms (see peak), but no peak is observed in the AV conditions (purple line in the same plot). The same scenario is evident for any other template map (Fig. 6*a,b*) of time windows 2 and 3.

Statistical analyses were run between AV and VA average fits to address whether the peak *R* values observed when fitting template maps to same-group conditions were significantly higher than any *R* value at any time point resulting from the fit to opposite-group conditions. A positive result would indeed indicate that each template map specifically belongs to one group of conditions, but not to the opposite group at any other latency. Using the same permutation-based approach as in our main analysis (maximum statistic; Nichols and Holmes, 2002), we compared via *t* tests the peak *R* value of each same-condition fit (e.g., purple peak in the leftmost plot in Fig. 6*b*) with every *R* value of its opposite-condition fit (e.g., with the green fit in the leftmost plot in Fig. 6*b*). The results of these between-conditions comparisons revealed that the peak correlation of each template map fit to same-group conditions was always significantly higher than any fit to the opposite-group conditions (Fig. 6*c*; *t* value represented by blue lines never go below the significance level α = 0.05, represented as purple and green lines). This demonstrates that the distinct patterns of EEG activity found for AV and VA simultaneity judgments in our main analysis are specific to each set of conditions and never emerge in the opposite conditions even when different latencies are taken into account. Therefore, AV and VA temporal binding are characterized by their own unique patterns of activity.

### AV and VA simultaneity judgments generate three distinct patterns of EEG activity over time

The fitting of template maps across conditions and latencies revealed an additional interesting aspect regarding the AV- and VA-specific EEG activity patterns. As illustrated in Figure 6*a*, map topographies not only differ between AV and VA clusters, but also across the time window of interest within AV and VA conditions. This is corroborated in Figure 6*b*, which shows that each of the AV and VA templates extracted from the 39–95 ms, 142–222 ms, and 297–351 ms time windows only peaks in its respective time window (highlighted by gray bars), not in any other time point. This would suggest that, when judging simultaneity of AV and VA stimuli, three distinct configurations of brain activity arise at three different latencies. To substantiate this further, we used the same approach as in the map latency analysis above, with the difference that statistical comparisons were made within and not between AV and VA conditions (Fig. 6*d*, within-conditions stats). Accordingly, we run *t* tests comparing the peak *R* value of each same-condition fit (e.g., purple peak in the leftmost plot in Fig. 6*b*) with all other *R* values of the same fit (e.g., other *R* values of the purple fit in the leftmost plot in Fig. 6*b*). Results revealed that each template map (Fig. 6*a*, bottom) significantly better fitted its specific time window compared with any other time point (Fig. 6*d*, *t* values represented by blue lines never go below significance level α = 0.05 for any time point outside of the time window of the template). This finding demonstrates that judging simultaneity of AV and VA events entails three different processing stages over time.

## Discussion

The results of our topographic ERP analyses shed new light on the neural underpinnings of audiovisual temporal binding. The multivariate analysis approach we used aimed to directly assess the (dis)similarity between brain responses under different experimental conditions, specifically testing whether instances of audiovisual temporal binding (indexed by high rates of audiovisual simultaneity perception across trials) are associated with brain activity in different networks according to the leading sensory modality. Although our method does not allow estimation of the underlying generators of ERPs recorded from the scalp, it represents a parsimonious way of addressing our specific research question (are AV and VA simultaneity judgments supported by the same brain network?) without relying on complex source localization procedures and additional data from other imaging techniques (e.g., MRI). Our results reveal consistently different activity maps between AV and VA ERPs after correction for their unisensory constituents, demonstrating that the evaluation of audiovisual simultaneity in the brain engages at least partially separate networks depending on the leading sense. In addition, we also found three distinct, consecutive multisensory brain activity patterns within each of these AV and VA networks, consistent with the idea that judging audiovisual simultaneity is a multistage process. These findings are discussed in detail below.

### Separate networks for audiovisual temporal binding depending on the leading sense

RSA (Kriegeskorte et al., 2008) has been used previously in M/EEG research to detect the emergence of activity patterns related to the encoding of specific stimulus features via comparison between the similarity structures of neural activations across stimulus categories (Carlson et al., 2011; Carlson et al., 2013; Cichy et al., 2014; Su et al., 2014; Zhang et al., 2015; Cichy et al., 2016a; Cichy et al., 2016b; Wardle et al., 2016). Here, we adapted this method to compare ERP topographies of multisensory interactions in conditions in which stimuli are identical (A and V) but their temporal order is varied (AV and VA). Using what we tentatively define as tRSA, we quantified (over a 500 ms window poststimulus) the similarity between the observed cross-correlation patterns of AV and VA ERP maps and two alternative cross-correlation models predicting either different (AV ≠ VA) or identical (AV = VA) maps for AV and VA processing. The results of our time-resolved tRSA showed that the AV ≠ VA model is the best fit to real data in three distinct time windows after the onset of the second stimulus of the audiovisual pair: 39–95 ms, 142–222 ms, and 297–351 ms. From a qualitative point of view, the topographies corresponding to each of these three time points show differences between AV and VA conditions, with AV maps presenting more right-lateralized activity and VA maps being generally more symmetrically organized. Importantly, these two specific sets of activity patterns corresponding to AV and VA processing are not a result of differences in ERP latencies because additional between-conditions analyses performed to address this concern showed that each AV and VA map only occurs within conditions of the same group and at a specific time point.

Our electrophysiological data corroborate recent behavioral evidence that AV and VA temporal binding are governed by independent processes, which have been suggested to involve efficient but nonmalleable mechanisms in the case of AV influences (e.g., visual attentional capture by sound) as opposed to VA interactions (e.g., speech comprehension enhancement by visual cues), which rely on temporally less accurate but highly flexible mechanisms (Cecere et al., 2016). Here, we show that these behaviorally separable AV and VA processes are also underpinned by distinct neural substrates, indicating that audiovisual interactions may require dedicated neural circuits and mechanisms based on the type of information available first (i.e., the leading sense). The existence of a dual route for evaluating audiovisual synchrony fits the most recent accounts proposing that different temporal binding windows and likely multiple binding mechanisms may be needed to accommodate different strategies for parsing information in primary auditory versus visual sensory systems (van Wassenhove, 2013; Thorne and Debener, 2014; VanRullen et al., 2014). According to theoretical models of auditory and visual sampling, the auditory and visual systems operate on different time scales and use different mechanisms to optimize sensory processing due to the intrinsic characteristics of each specific class of stimuli that they process. More specifically, whereas the auditory system may rely on multifrequency sampling (from low-delta to high-gamma frequencies) (Poeppel, 2003; Giraud and Poeppel, 2012; Gross et al., 2013) and temporal prediction (Arnal and Giraud, 2012; Stekelenburg and Vroomen, 2012) to capture the rapid changes in auditory signals, the visual system is thought to use a relatively low sampling rate and to scan the environment periodically to acquire visual information that is more stationary in nature (VanRullen and Koch, 2003; Busch et al., 2009; Busch and VanRullen, 2010; Schroeder et al., 2010; VanRullen et al., 2014). Our data would suggest that crossmodal information also may serve different purposes within each class of multisensory interactions (AV vs VA), potentially building on the fundamental differences between auditory and visual processing outlined above and, consequently, that crossmodal influences may be achieved through more diversified and dynamic mechanisms than was previously thought.

One candidate substrate to bind information flexibly across networks is synchronized oscillatory brain activity, as suggested by a growing number of studies (Schepers et al., 2013; Gleiss and Kayser, 2014a, 2014b; van Atteveldt et al., 2014; for reviews, see: Engel et al., 2012; Senkowski and Engel, 2012; Cecere et al., 2015; Park et al., 2016). This view is supported by evidence that inputs in one sensory modality can influence stimulus processing directly in other primary sensory areas by phase resetting ongoing oscillations (Lakatos et al., 2007; Kayser et al., 2008; Fiebelkorn et al., 2011; Naue et al., 2011; Thorne et al., 2011; Diederich et al., 2012; Romei et al., 2012; Mercier et al., 2013). Based on these findings, some investigators have suggested that crossmodal interactions might be driven by different phase-resetting mechanisms depending on the leading sensory modality, with anticipatory auditory and visual cues serving different functions. In such models, leading auditory information can serve as an alerting mechanisms boosting visual processing in AV interactions, whereas leading visual input may help the auditory system in generating predictions of upcoming auditory signals in VA interactions (Thorne and Debener, 2014). Although our findings do not allow distinguishing these accounts, they do support the notion of distinct networks and binding mechanisms being at play for AV and VA interactions.

### Evaluation of audiovisual simultaneity as a multistage process

In addition to the evidence of different networks being engaged in AV and VA simultaneity judgments, our analyses revealed a significantly different set of AV- and VA-specific ERP maps in the course of multisensory processing (i.e., in the 39–95 ms, 142–222 ms, and 297–351 ms windows poststimulus-2 onset). The early differences (39–95 ms) that we found between AV and VA topographies are consistent with previous studies using synchronous audiovisual pairs and reporting early multisensory effects starting at ∼50 ms poststimulus onset that are generally characterized by a parieto-occipital positivity in the difference ERP maps (Giard and Peronnet, 1999; Fort et al., 2002; Molholm et al., 2002; Vidal et al., 2008; Cappe et al., 2010; Cappe et al., 2012; Murray et al., 2016). Although they confirm that multisensory interactions in general are very fast processes, our results also extend these previous findings by showing that different multisensory networks may be recruited as early as ∼40 ms poststimulus onset based on the temporal order of auditory and visual inputs.

Likewise, the later differences between AV and VA activation patterns that we observed (142–222 and 297–351 ms) are consistent with previous ERP studies that implemented the additive model to characterize crossmodal interactions (Senkowski et al., 2007; Vidal et al., 2008). Of particular interest is the study by Senkowski et al. (2007), which analyzed ERPs in response to either naturalistic or non-naturalistic audiovisual pairs where the visual constituents always preceded the sound (i.e., corresponding to VA conditions in our experiment). Interestingly, not only the time windows of the late activations reported by Senkowski et al. (2007) (210–250 ms and 300–350 ms postsound) largely overlap with those that we found, but also the corresponding difference maps (see: non-naturalistic maps of Fig. 5 in Senkowski et al., 2007) are strikingly similar to our VA maps (cf. green-bordered maps of time windows 2 and 3 in our Fig. 6*a*), with posterior positivity at ∼200 ms and central-posterior positivity at ∼300 ms after sound onset [please note that positive and negative activations have to be considered inverted in Fig. 5 of Senkowski et al., 2007 because they used (A+V) − AV instead of AV − (A+V) to compute the difference maps].

We interpret this posterior-to-anterior shift of activity over time, which we observed for both AV and VA stimuli (Fig. 7), to represent three processing stages leading to perception of audiovisual synchrony, likely based on succesful temporal binding at early stimulus processing stages and decisional processes at a later stage. Importantly, the observation that each processing stage is characterized by different topographies between AV and VA conditions suggests that the three-step process to evaluate audiovisual simultaneity unfolds through different networks based on the leading sense.

### Conclusion

In this ERP study, we used a novel topographical analysis approach to investigate the networks underlying temporal integration of AV and VA audiovisual stimuli. Our findings were twofold. First, we found that separate networks are recruited selectively for evaluating audiovisual synchrony depending on the leading input (auditory or visual), suggesting that temporal binding of audiovisual information requires multiple and flexible mechanisms. In addition, each of these separate networks for AV and VA processing was characterized by the emergence of three distinct brain states over time, configuring AV and VA simultaneity perception as multistage processes. The recruitment of multiple networks and mechanisms during the evaluation of audiovisual synchrony may represent a flexible solution to maximize the benefit of crossmodal cues in each sensory system and support different cognitive operations such as auditory boost of visual attention or visual enhancement of speech comprehension.
